# Protein lactylation: a metabolic signal driving cancer therapy resistance

**DOI:** 10.1038/s41420-026-03050-w

**Published:** 2026-03-31

**Authors:** Silvia D’amico, Sara Giovannini, Gerry Melino, Angelo Peschiaroli, Francesca Bernassola

**Affiliations:** 1 Institute of Translational Pharmacology (IFT), CNR, Rome, Italy; 2 Department of Experimental Medicine, TOR, University of Rome Tor Vergata, Rome, Italy

**Keywords:** Cancer metabolism, Cancer therapeutic resistance

## Abstract

Unlike normal cells, which primarily rely on oxidative phosphorylation, cancer cells reprogram their metabolism by preferentially utilizing glycolysis even in the presence of oxygen to generate ATP. As a result, cancer cells and the tumor microenvironment typically accumulate high levels of lactate. Although initially considered a mere byproduct of glucose metabolism, lactate has recently emerged as an important metabolic intermediate involved in many intracellular pathways and protein modifications. Lysine lactylation is indeed a newly identified, metabolism-linked post-translational modification in which lactate is covalently bound to specific lysine residues. This review provides an overview of the current understanding of how lysine lactylation mechanistically contributes to therapeutic resistance in tumor cells. Remarkably, protein lactylation is emerging as a promising druggable approach for overcoming therapy resistance. Hence, here, we also highlight new strategies that target lactylation with pharmacological inhibitors to counteract drug resistance in cancer.

## Facts

- The metabolic rewiring of cancer cells drives lactate accumulation.
- Lactate acts as a substrate for protein lysine lactylation, linking metabolism to epigenetic, transcriptional and post-translational regulation.
- Histone lactylation modulates chromatin accessibility and transcription of genes involved in DNA damage repair.
- Lactylation can also affect DNA damage repair capacity through direct modification of proteins implicated in multiple repair pathways.
- Lactylation modulates the expression of immune checkpoints, macrophage polarization and myeloid function.
- Overall, lactylation reduces the efficacy of anticancer treatments, including chemo-, radio- and immunotherapy.

## Open questions

- What is the full repertoire of Kla-regulating enzymes?
- How does lactylation interact/compete with other post-translational modifications to regulate therapeutic responses?
- Can protein lactylation serve as a predictive or prognostic biomarker for therapy responses?
- Can therapeutic modulation of lactylation provide an alternative strategy to overcome cancer therapy resistance?

## Introduction

The rapid proliferation of cancer cells requires a profound metabolic reprogramming characterized by a strategic shift from energy-efficient catabolism to a growth-oriented anabolic program. While normal tissues primarily rely on mitochondrial oxidative phosphorylation (OXPHOS) for energy production, neoplastic cells often prioritize glycolysis, even under normoxic conditions [1]. While retaining functional mitochondrial OXPHOS, this phenotype, known as the Warburg effect [2], reflects the need for rapid adaptation. Indeed, even though it is less efficient per glucose molecule, glycolysis generates ATP much faster than OXPHOS. Beyond ATP production, the breakdown of glucose through glycolysis provides essential carbon intermediates that fuel macromolecule biosynthesis [1]. For instance, glucose-6-phosphate can be shunted into the pentose phosphate pathway to generate ribose for nucleotide biosynthesis, while simultaneously generating NADPH to support reductive reactions including fatty acid synthesis. Additional anabolic precursors derived by glycolysis include fructose-6-phosphate that contributes to glucosamine-6-phosphate synthesis for glycosaminoglycan production, and dihydroxyacetone phosphate that serves as a source of glycerol for tryglyceride synthesis. Importantly, 3-phosphoglycerate can feed into the serine biosynthetic pathway. Serine eventually supports several biosynthetic and redox functions, including nucleotide and lipid synthesis, and NADPH generation. By providing intermediates for the biosynthetic pathways and biomass accumulation, these metabolic adaptations of cancer cells sustain their rapid cell growth and division.

Because of this metabolic rewiring, cancer cells typically display the accumulation of abnormally high levels of lactate with consequent acidification of the tumor microenvironment (TME) and immunosuppression [3]. Why and how is the Warburg effect regulated? The question is crucial as it provides a significant amount of lactic acid, however this is not an easy question; indeed, it is still wide open after a century of research [2]. A possible mechanism is related to the detachment of hexokinase type 2 (HK2) from the outer mitochondrial membrane, thereby driving sufficient glycolytic ATP production [4]. Hence, HK2 seems to be essential to regulate compartmentalized ATP synthesis and aerobic glycolysis. An alternative mechanism seems to involve the polyol pathway that allows the transformation of glucose into fructose via the AKR1B1 and SORD enzymatic action, thus overcoming the regulatory action of HK and phosphofructokinase [5, 6]. This mechanism has been described both for pancreatic [7] and colorectal [8] cancers.

Advances in metabolomics have revealed that selected metabolic intermediates have unexpected secondary functions [1]. Depending on their availability, certain metabolites can modify gene expression, protein function, and the epigenetic profile of the cell, ensuring a rapid adaptive response to changes in energy demand [1]. One common mechanism is metabolite-induced post-translational modifications (PTMs), which involve the transfer of functional groups from metabolic intermediates to amino acid residues within proteins [9]. Based on the functional group donor, PTMs can occur enzymatically or non-enzymatically and include, among others, acetylation, methylation, succinylation, and glycosylation [9].

Recently, a new PTM has been described in which lactyl groups are covalently attached to lysine (Lys, K) residues [10]. Known as Lys-lactylation (hereafter referred to as Kla), it was first identified as a modulator of gene transcription through histone modification and subsequent reshaping of chromatin structure, thereby linking metabolic reprogramming to epigenetic regulation [10]. In keeping, Kla is now evident as key player in colorectal cancer [11], glioblastoma [12] as well as astrocytes [13] or macrophage polarization [14]. In the last years, several non-histone proteins have been identified as targets of Kla [15], revealing a crucial multifaceted role of this PTM in cancer biology. From this new perspective, lactate, previously regarded as a mere byproduct of glycolysis, is now emerging as a signaling molecule [16, 17] that links the metabolic reprogramming of cancer cells to transcriptional and post-transcriptional regulation.

Kla has been described as implicated in a wide range of cancer cell functions, including tumor proliferation, metastasis, maintenance of stemness, immune evasion, and therapeutic resistance [18]. This review will focus on the role of Kla in the establishment of therapeutic resistance, with a particular attention to emerging strategies that target Kla to counteract drug resistance in cancer.

## Biochemical mechanisms of protein lactylation

**Fig. 1:**
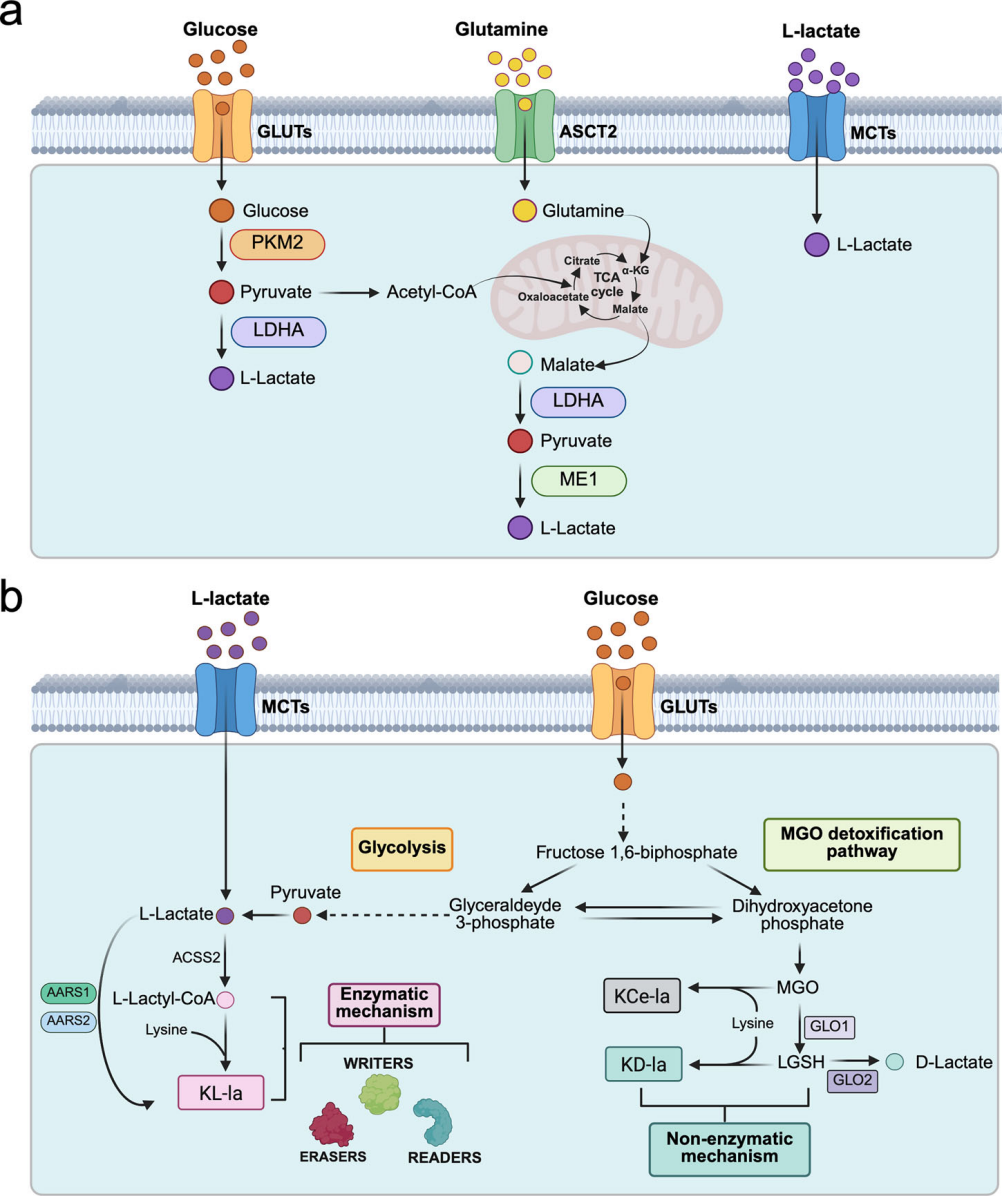
Lactate production in tumor cells and mechanisms of protein lactylation. **a** Primary sources of lactate in tumor cells. (1) Glucose, transported into the cell via glucose transporters (GLUTs), is metabolized through glycolysis to generate pyruvate, which is subsequently converted to L-lactate by LDHA. As a less utilized route, pyruvate can be converted into acetyl-CoA and enter the tricarboxylic acid (TCA) cycle, where it condenses with oxalacetate to generate citrate and support mitochondrial oxidative metabolism. (2) Glutamine, transported into cells via the alanine/serine/cysteine transporter (ASCT2), is metabolized in the mitochondria to glutamate, which is then converted to α-ketoglutarate (α-KG) that enters the TCA cycle to produce malate. In the cytosol, malate is converted to pyruvate, which is subsequently reduced to L-lactate. (3) MCT transporters facilitate the bidirectional exchange of lactate between tumor cells and the TME **b** Schematic representation of the enzymatic and non-enzymatic mechanisms of Kla. In the enzymatic pathway, lactyl-CoA synthetase (ASS2) converts L-lactate into L-lactyl-CoA, whose lactyl group is then transferred to Lys residues on target proteins by specific lactyl transferases (“writers”). The removal and recognition of the lactyl moiety on target proteins involve the enzymatic action of “eraser” (deacetylase) and “reader” proteins, respectively (see text for detail). KL-La can also occur directly using L-lactate as the substrate via the enzymatic activity of AARS1 and AARS2 enzymes. The non-enzymatic mechanism of K-la involves the direct transfer of the lactyl moiety from LGSH, an intermediate metabolite generated by GLO1 within the MGO detoxification pathway, to the target proteins. The highly reactive metabolite MGO can also react with a variety of protein residues, including Lys, generating the N-ε-(carboxyethyl)-lysine modification (Kce-la) on target proteins.

L-lactyl-lysine (K_L-la_) typically arise in response to enhanced glycolytic activity, glutamine metabolism, or hypoxic conditions [10, 19, 20]. As part of their metabolic reprogramming, cancer cells preferentially reduce pyruvate to L-lactate, shifting from OXPHOS to glycolysis to meet their energy and biosynthetic demands, even under aerobic conditions [1] (Fig. 1a). Glutamine, whose uptake is aberrantly increased in cancer cells, can be enzymatically converted into pyruvate and ultimately L-lactate, further contributing to the intracellular lactate pool (Fig. 1b). In addition to their own metabolic activity, tumor cells utilize L-lactate produced within the TME by neighboring cells, including Warburg-dependent cancer cells, and cancer-associated fibroblasts [21] (Fig. 1a). Consequently, L-lactate is the main and most abundant byproduct of glycolysis in cancer cells, with a concentration typically ranging from 10 to 30 mM [10], and K_L-la_ represents the primary form of Kla within the cell [19].

Beyond K_L-la_, further studies have revealed the existence of additional Kla isomers with identical molecular weight and highly similar structures, including D-lactyl-lysine (K_D-la_) and N-ε-(carboxyethyl)-lysine (K_ce-la_) (Fig. 1b) [19, 22, 23].

Reverse HPLC can identify K_ce-la_, while K_L-la_ and K_D-la_ differ in configuration at a single stereogenic center, thus requiring a chiral derivatization reaction to be resolved [19]. Despite their structural similarities, these PTMs differ in terms of their metabolic origin and function.

In the main L-Kla pathway, lactyl-CoA synthetase converts L-lactate into L-lactyl-CoA [24], whose lactyl group is then transferred to Lys residues on target proteins by specific lactyl transferases [22, 24] (Fig. 1b). As with other PTMs, protein Kla can implicate the activity of “writer” (lactyltransferases) and “eraser” (delactylases) and “reader” enzymes that are responsible for the addition, removal and recognition of the lactyl moiety, respectively (Fig. 1b). To date, no enzymes with exclusive lactyl transferase activity have yet been identified. However, due to structural similarity between lactate and acetyl groups, certain histone acetyltransferases, have been suggested to act as L-Kla “writers”. These include CREB-binding protein (CBP), E1A-binding protein p300 (EP300), K acetyltransferase (KAT) 2 A, KAT5 (TIP60), KAT7, and KAT8 [22]. Since Kla and acetylation share not only the same “writers” but also the same target residues, it is not surprising that these PTMs can compete for modification of the same Lys residues, thus adding a layer of regulation to the epigenetics of the cell [24].

Alternatively, alanyl-tRNA synthetase 1 (AARS1) and alanyl-tRNA synthetase 2 (AARS2), enzymes that typically catalyze the attachment of L-alanine to tRNAs during protein synthesis, can directly promote protein Kla using lactate, rather than lactyl-CoA, as the substrate [24, 25] (Fig. 1b). In this reaction, lactate binds to AARS1/2 in an ATP-dependent manner to form a lactate-AMP intermediate, which, subsequently, transfers the lactyl group to Lys residues on target proteins.

K_D-la_ and K_ce-la_ are generated non-enzymatically through the reaction of the ε-amino group of the Lys side chain with two intermediates associated with high glycolytic flux: methylglyoxal (MGO) and S-D-lactylglutathione (LGSH) (Fig. 1b) [26, 27]. MGO, a highly reactive molecule derived from the fragmentation of triose phosphate, can spontaneously, aspecifically, and irreversibly modify proteins, including through K_ce-la_ [26]. The biological significance of this PTM is unclear, though it is associated with diabetes and aging and affects the stability of target proteins [28].

LGSH is generated from MGO through a detoxifying reaction catalyzed by glyoxalase 1 (GLO1) within the glyoxalase pathway (Fig. 1b). LGSH is then hydrolyzed by glyoxalase 2 (GLO2), yielding D-lactate, or it can modify Lys residues to form K_D-la_ [23, 27]. Recently, the D-lactyl moiety seems also to be transferred non-enzymatically from LGSH to CoA, resulting in the formation of D-lactyl-CoA [27]. Although under physiological conditions, L-Kla represents the predominant form of Kla [19], perturbations in the glycolytic flux or in the MGO detoxification pathway can lead to abnormal LGSH accumulation and a consequent increase in D-Kla events. Consistent with this notion, genetic ablation of the LGSH-converting enzyme GLO2 [27] or its downregulation in response to inflammatory stimuli [29] results in marked LGSH accumulation with the concomitant increase of K_D-la_. These findings indicate that the non-enzymatic pathway might be an additional regulatory mechanism connecting glycolytic activity with protein function via Kla.

Since the primary function of Kla is to enable cells to rapidly adapt to metabolic changes, this PTM must be dynamic. This means that it is reversible and can be removed by “eraser” proteins.

Although no enzymes with selectivity for lactyl residues have been identified, it has been shown that histone deacetylases and sirtuins can also hydrolyze lactyl-Lys bonds, thereby restoring unmodified Lys residues. Interestingly, these enzymes do not exhibit stereospecificity, acting on both the L- and D-enantiomers of lactyl-Lys [30–32]. On the other side, few “reader” enzymes have been characterized, including the TRIM33 PHD-bromodomain [33], Brahma-related gene-1 [34], and decreased expression in fat 2 [35] being the only ones identified to bind L-lactylated histones. Interestingly, a study on the effects of D-Kla on the inflammatory signaling of macrophages indicated the presence of a potential KD-la reader belonging to the BET family of bromodomain-containing proteins [27].

## Outcomes of protein lactylation

Histone Kla generally promotes chromatin decondensation by either altering the nucleosome surface charge or recruiting specific “reader” proteins that may facilitate chromatin opening, thereby positively regulating gene expression. However, Kla of histone Lys does not universally promote transcription. In fact, some studies have shown that histone Kla can also contribute to gene repression, depending on the site and context. Histone Kla has been detected across all four core histones (H2A, H2B, H3, H4). However, the most prominent and functionally characterized sites are H3 Lys18 (H3K18la), H3 Lys9 (H3K9la), and H4 Lys12 (H4K12la) [36].

The effect of Kla is rather complex and pleotropic, including for example mitophagy [37] or acetylcytidine modifications at the level of tRNAs [38]. At the non-histone level, the covalent addition of a lactate moiety to Lys residues can modify protein conformation or charge, thereby influencing their biological activity, stability and subcellular localization. In addition, Kla introduces a chemical group capable of forming hydrogen bonds, which can influence protein-protein and protein-DNA interactions.

Kla can modulate both the catalytic activity and subcellular localization of metabolic enzymes involved in diverse metabolic pathways, including glycolysis, the TCA cycle, and fatty acid and nucleotide metabolism. Several glycolytic enzymes, such as glyceraldehyde-3-phosphate dehydrogenase (GAPDH), fructose-bisphosphate aldolase A (ALDOA), and pyruvate kinase M2 (PKM2) are lactylated, with different effects on their enzymatic activities [39]. For example, Kla of ALDOA alters its subcellular localization and inhibits its enzymatic activity, thereby suppressing the glycolytic flux [39]. In contrast, Kla of PKM2 enhances its kinase activity and promotes its nuclear localization [40].

In addition to glycolysis, Kla also impacts the TCA cycle. Kla of key mitochondrial enzymes, including pyruvate dehydrogenase E1 subunit alpha 1 (PDHA1) and carnitine palmitoyltransferase 2 (CPT2), suppresses their enzymatic activities, leading to inhibition of the TCA cycle and a consequent reduction in OXPHOS [41].

Notably, an integrative lactylome and proteome analysis in hepatocellular (HCC) carcinoma revealed that the Kla of adenylate kinase 2, a critical enzyme catalyzing the conversion of ATP into ADP, reduces its enzymatic activity and promotes tumor cell proliferation [42].

Collectively, these lines of evidence indicate that Kla of key metabolic enzymes, likely through the enzymatic mechanisms, establishes a feedback loop, in which metabolic reprogramming and Kla reciprocally influence each otherr.

The non-enzymatic pathway of Kla has also been involved in the modulation of cellular metabolism. Global D-lactylome profiling in GLO2-deficient cells has revealed that D-Kla of glycolytic enzymes establishes a feedback mechanism that restrains the glycolytic flux under conditions of elevated LGSH [23]. Beyond the glycolytic metabolism, D-Kla also regulates de novo serine biosynthesis, a pathway essential for one-carbon metabolism, glutathione production, and nucleotide biosynthesis, which are required to sustain cancer cell proliferation [43]. Specifically, in GLO2-deficient cells, D-Kla modification of phosphoglycerate dehydrogenase, the first and rate-limiting enzyme in de novo serine biosynthesis, leads to the inhibition of its enzymatic activity, thereby resulting in reduced intracellular levels of serine [43]. Collectively, these findings indicate that Kla is dynamically regulated to reprogram metabolic pathways in response to cellular metabolic changes and specific metabolic needs.

Of note, metabolic reprogramming has emerged as a central mechanism driving chemoresistance in cancer cells. Among the metabolic pathways implicated, Kla has garnered attention as a crucial regulator of chemoresistance, particularly in tumors exhibiting high glycolysis and lactate production. Recent evidence indeed highlighted how Kla strengthens DNA repair mechanisms, enabling cancer cells to survive genotoxic stress and impairing the efficacy of chemotherapy and radiotherapy.

## Implications of protein lactylation in tumor therapy resistance

Altered metabolism and genome instability are fundamental hallmarks of cancer. Growing evidence highlights that these processes are tightly functionally interconnected. Indeed, the primary mechanism of action of radiotherapy and chemotherapeutic drugs is the induction of DNA damage to promote cell death. Consequently, to survive these insults, cancer cells depend on rapid and efficient DNA repair pathways. Importantly, they harness specific metabolites to support and enhance DNA damage repair. Among these metabolites, lactate is emerging as a key regulator of both histone and non-histone protein modification, thereby contributing to the development of therapeutic resistance [44].

Double-strand breaks (DSBs), the most deleterious DNA lesions elicited by radio- and chemotherapy, are primarily repaired through two pathways, non-homologous end-joining (NHEJ) and homologous recombination (HR) [45, 46]. Single-strand breaks (SSBs) induced by oxidizing and alkylating agents can be fixed by the base excision repair (BER)/SSB repair pathway [47].

In the HR repair system, the MRN complex, composed of MRE11 (meiotic recombination 11), NBS1 (Nijmegen breakage syndrome 1), and RAD50, binds and cleaves DNA in cooperation with another structure-specific nuclease, CtIP (C-terminal binding protein (CtBP)-interacting protein), to initiate end resection [48, 49]. Importantly, the DNA binding and nuclease functions of MRE11 are regulated by NBS1, which is necessary for MRN role in HR activation [50, 51]. Upon NBS1 binding, MRN targets ATM to DSBs. Subsequent ATM activation and ATM-dependent phosphorylation of H2AX (γH2AX) further signals the presence of DNA damage and triggers HR-dependent repair [51]. The exposure of a ssDNA induces the recruitment of replication protein A (RPA), to coat and protect it from degradation [52]. RPA also stimulates the exonucleases EXO1 and DNA2 for long-range resection [53], with the help of the BRCA1 (breast cancer protein 1)/BRCA2 (breast cancer protein 2)/BARD1 (BRCA1-associated RING domain 1) complex [54]. HR further proceeds with the replacement of RPA by RAD51 nucleoprotein filament [52], which finally induces the homology search for recombination [45]. The strand invasion generates a recombination intermediate, which is subsequently processed by Bloom (BLM) helicase in complex with topoisomerase IIIα (TOP3A), RMI1 and RMI2 (RecQ-mediated genome instability proteins 1 and 2), to finally form non-crossover products, thereby maintaining genome stability [55, 56].

While HR operates in the G2/S phase, when replication provides sister chromatids for the repair process, NHEJ is active throughout the cell cycle, particularly in G1. Here, the DSB repair pathway choice favors the activity of 53BP1 (p53 binding protein 1) in counteracting BRCA1-dependent end resection, to conversely facilitate NHEJ [54, 57, 58].

In this pathway, DNA lesions are bound by the Ku70-Ku80 heterodimer, which recruits the catalytic subunit of the DNA-PK (DNA-protein kinase) complex (DNA-PKcs), whose regulatory kinase activity promotes the recruitment of the downstream factors [46]. DNA-PK interacts with the X-ray repair cross-complementing protein 4 (XRCC4)/ligase IV complex at DNA termini, thus stimulating its ligase activity [59]. Importantly, upon Kla [60], the other main scaffold protein of this pathway, XLF (XRCC4-like factor), helps in stabilizing the complex by interacting with Ku80 [61]. Moreover, XLF facilitates XRCC4 binding to DNA. In a complex, they form mobile sleeve-like structures around broken DNA molecules to bridge them [62, 63] and favor the final ends ligation step.

Interestingly, DSBs can also arise from collapse of replication forks encountering unrepaired SSBs, which may occur spontaneously, be induced by radiation, or arise as obliged BER intermediates [64, 65]. BER is primarily activated by DNA base modifications, induced by oxidizing or alkylating agents, which are recognized and removed by specific enzymes.

As an initial step, BER glycosylases cleave the glycosidic bond, generating an abasic site, that subsequently stimulates endonuclease-dependent incision of the DNA sugar-phosphate backbone [66]. Consequently, the resolution of the arising SSB intermediate involves both BER and SSBR downstream factors [67], such as XRCC1 (X-ray repair cross-complementing protein 1) [68–70]. This scaffold protein drives the final DNA break sealing by stabilizing DNA polymerase β and ligase III [70, 71].

Additionally, once recruited by PARP1 (poly(ADP-ribose) polymerase 1) on the sites of DNA breaks [69, 72], XRCC1 undergoes different PTMs, such as phosphorylation, ubiquitination and Kla [73, 74], regulating its fundamental function in SSBs and DSBs repair.

Given that genomic instability is a hallmark of cancer cells, they counteract the accumulation of DNA damage by potentiating DNA repair pathways. However, overactivation of these repair mechanisms can enhance repair capacity, and ultimately lead to therapy resistance in cancer cells.

### Histone lactylation in therapy resistance

**Fig. 2:**
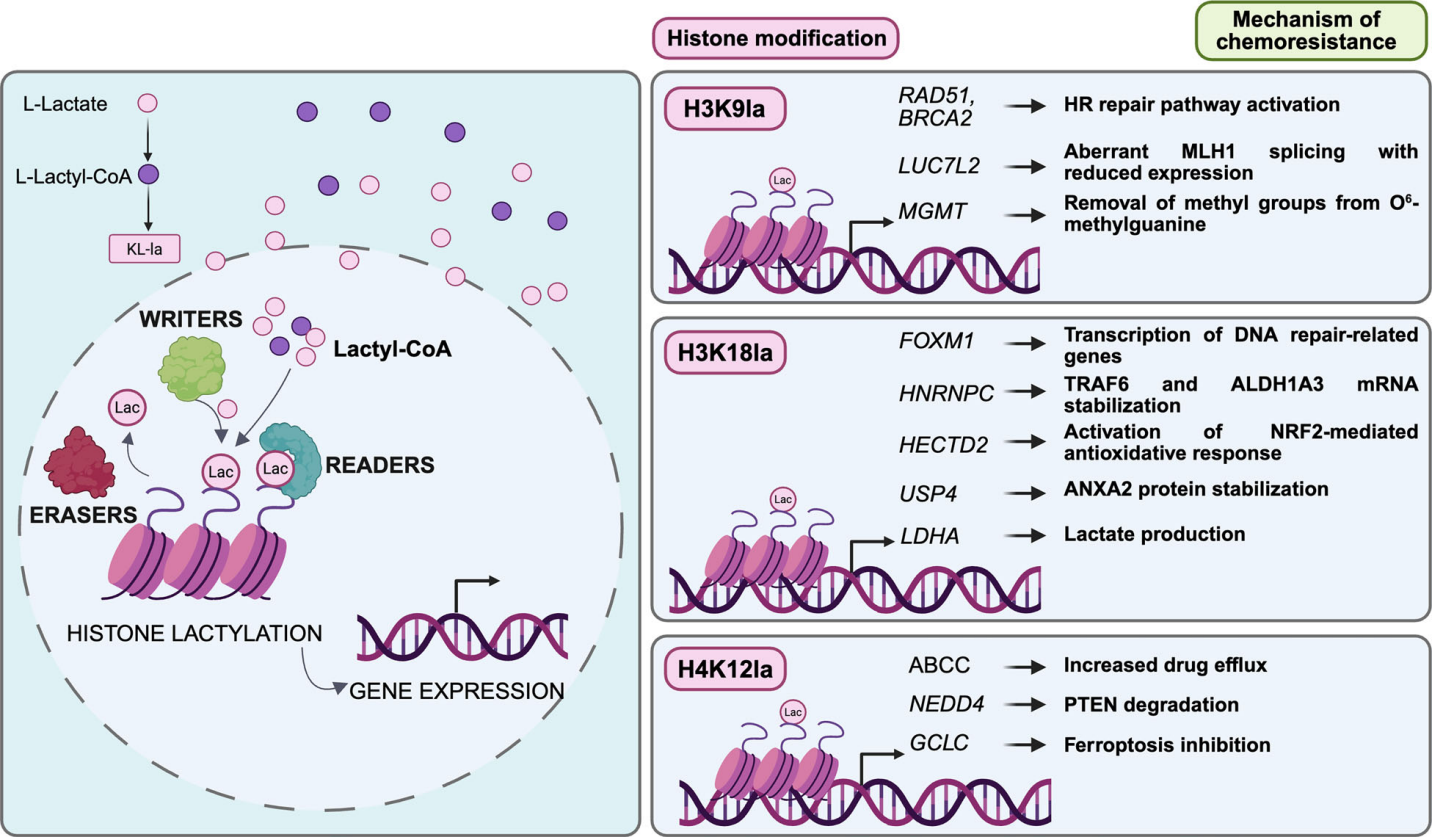
Mechanistic roles of histone Kla in therapy resistance. Histones Kla induces structural changes in chromatin that enhance the accessibility of DNA to transcription factors, leading to increased gene expression. As for other PTMs, histone Kla involves “writers” (lactyl transferases) that add lactyl groups, “erasers” that remove them, and “readers” recognizing these marks to regulate transcription. Kla can promote therapeutic resistance by modulating histone modifications, thereby activating the transcription of genes encoding key regulators of DNA damage repair pathways. This figure summarizes the predominant histone Kla sites (H3K18la, H3K9la, and H4K12la), the substrates modified by each histone modification, and links Kla on specific substrates to the molecular mechanisms underlying therapy resistance.

One of the mechanisms by which Kla promotes therapeutic resistance is through indirectly inducing histone modification to activate transcription of genes encoding proteins essential for DNA damage repair regulation [75] (Fig. 2). Yue and colleagues reported that H3K9la confers temozolomide (TMZ) resistance of glioblastoma (GBM) by upregulating LUC7L2 transcription [75]. LUC7L2, an RNA-binding protein, acts as a splicing factor that facilitates intron 7 retention in the MLH1 mRNA, leading to reduced MLH1 expression, and subsequent suppression of the mismatch repair (MMR) pathway. Stiripentol, an anti-epileptic drug known to inhibit LDHA and to cross the blood–brain barrier, was shown to lower H3K9la thereby enhancing the responsiveness of GBM cells to TMZ. Collectively, these findings delineate one of the mechanisms through which Kla drives TMZ resistance in GBM and underscore the potential of Kla inhibition as a therapeutic strategy to counteract treatment failure.

H3K9 modification has been associated with GBM chemoresistance to TMZ via an alternative mechanism involving Sirtuin 6, an eraser of H3K9 Kla [76]. By reducing H3K9 Kla, Sirtuin 6 suppresses the expression of O^6^-methylguanine-DNA methyltransferase (MGMT), a key determinant of TMZ therapeutic efficacy. MGMT drives resistance by removing DNA-damaging methyl groups introduced by the drug. Of note, TMZ-resistant GBM cells exhibit decreased nuclear SIRT6, resulting in elevated MGMT levels and enhanced chemoresistance. The authors demonstrated that pharmacological activation of SIRT6 significantly sensitizes GBM cells to TMZ, underscoring its potential as a therapeutic target to enhance TMZ effectiveness in GBM therapy.

In a recent study [77], Sun and co-authors performed gene set enrichment analysis to identify changes in Kla between platinum-resistant and platinum-sensitive ovarian cancer patients. The significantly higher expression of glycolytic genes in the resistant group suggested histone Kla as driver of resistance. The authors confirmed higher pan-Kla levels in cisplatin-resistant ovarian cancer cells, with prominent signal intensity in the histone region, indicating H3K9 as the mostly increased Kla histone site. Accordingly, the authors reported a significant increase in the glycolytic rate, glucose uptake, and lactate production in cisplatin-resistant cells. Additionally, they identified GCN5 as the writer enzyme of H3K9la, which ultimately stimulates the expression of the HR factors, RAD51 and BRCA2 (see next paragraph) [77]. By performing ChIP-qPCR, the authors demonstrated that treatment with lactate or glycolysis inhibitors could modulate the enrichment of H3K9la at the promoter regions of RAD51 and BRCA2 [77]. The observed Kla-dependent enhancement of HR consequently conferred resistance, which is indeed associated with poor prognosis in ovarian cancer.

Sheng and colleagues reported that upregulation of H3K18la is a hallmark of docetaxel (DTX)-resistance in prostate cancer cells [78]. In DTX-resistant prostate cancer cells, H3K18la activates the expression of FOXM1, a transcription factor, whose overexpression correlates with therapy resistance [79, 80]. Furthermore, H3K18la transcriptionally activates the expression of heterogeneous nuclear ribonucleoprotein C (HNRNPC), which acts as an m6A “reader” to stabilize TNF receptor-associated factor 6 (TRAF6) mRNA, ultimately promoting TRAF6-mediated gemcitabine resistance in pancreatic ductal adenocarcinoma (PDAC) [81]. Through a similar m6A-dependent mechanism, HNRNPC stabilizes aldehyde dehydrogenase 1 family member A3 (ALDH1A3) mRNA, a positive regulator of glycolysis. This, in turn, increases lactate production, reinforcing H3K18la and creating a positive feedback loop that sustains HNRNPC’s expression. Coherently, pharmacological inhibition of ALDH1A3 was able to overcome gemcitabine resistance in PDAC cells, suggesting that targeting the HNRNPC-ALDH1A3 axis could offer a novel therapeutic approach for PDAC patients.

H3K18la also contributes to drug resistance in HCC, as exemplified by reduced sensitivity to lenvatinib [82]. In HCC, H3K18la drives the upregulation of HECTD2 in lenvatinib-resistant cancer cells. HECTD2 is an E3 ubiquitin ligase that targets, among other protein substrates, the Kelch-like ECH-associated protein 1 (KEAP1). Ubiquitination and subsequent degradation of KEAP1 by HECTD2 activate the NRF2-mediated antioxidative response, which lowers cellular ROS and diminishes the sensitivity of HCC cells to Lenvatinib [82].

H3K18la is also responsible for the upregulation of USP4, a deubiquitinase overexpressed in GBM stem cells, where it is implicated in the maintenance of stemness and radioresistance through the USP4/Annexin A2 (ANXA2) axis [12]. Specifically, USP4 interacts with ANXA2, resulting in its stabilization via deubiquitination and consequent protection from proteasomal degradation. The USP4/ANXA2 axis, in turn, promotes therapeutic resistance in GBM by inducing bone marrow X-linked kinase-mediated activation of STAT3.

An interesting study pointed out histone Kla as a triggering process for lung cancer resistance to epidermal growth factor receptor tyrosine kinase inhibitors (EGFR-TKI) [83]. Resistance was found to be driven by the over-expression of collagen triple helix repeat-containing 1 (CTHRC1) in a subpopulation of CAFs. The authors reported that CTHRC1-positive CAFs are markedly enriched in drug-resistant lung tumors and critically contribute to metabolic reprogramming in cancer cells. In particular, CTHRC1 drives the upregulation of hexokinase II, thereby enhancing the glycolytic activity of cancer cells through the activation of the TGF-β/SMAD3 pathway. The excess of lactate produced during glycolysis then boosts CTHRC1 transcription in CAFs through H3K18la, forming a CTHRC1/glycolysis/H3K18la positive feedback loop that sustains EGFR-TKI resistance [83].

Similarly to H3K18la, H3K14la is involved in histone modification-mediated drug resistance. In HCC, this modification drives resistance to oxaliplatin via the transcriptional upregulation of Neuronal precursor cell-expressed developmentally downregulated 4 (NEDD4) [84]. Importantly, this E3 ubiquitin ligase exerts its function on multiple substrates, involved in DNA damage response, contributing to tumorigenesis [85, 86]. In the study by Zeng and colleagues, H3K14la enhances NEDD4 levels to target PTEN for subsequent degradation, thereby inducing the activation of PI3K/Akt/mTOR signaling pathway [84]. PTEN degradation was accompanied by enhanced glycolysis that ultimately led to the development of resistance to 5-fluorouracil and oxaliplatin in HCC cells and to the establishment of a glycolysis/H3K14la/PTEN positive feedback loop [84]. These findings interestingly demonstrate the involvement of histone Kla in another positive feedback loop contributing to cancer resistance to therapies.

Another study demonstrated that high Kla levels correlate with a poor prognosis in patients with colorectal cancer (CRC) [87]. Specifically, p300-mediated Kla of H4K12 fosters the transcription of glutamate-cysteine ligase (GCLC), an enzyme involved in glutathione synthesis that prevents ferroptosis by inhibiting lipid peroxidation. Histone H4 modification drives GCLC upregulation, thereby enhancing chemoresistance through ferroptosis suppression. Of note, treatment with oxamate, an LDHA inhibitor, or targeting GCLC with the BSO inhibitor decreased chemoresistance.

H4K12la has also been implicated in regulating the transcription of the ABC transporters ABCC2, ABCC3, and ABCC10 through modification of their gene promoters [88]. The resulting increases in drug efflux reduce the chemosensitivity of colorectal cancer cells.

### Lactylation of regulators and effectors of DNA damage repair in therapy resistance

**Fig. 3:**
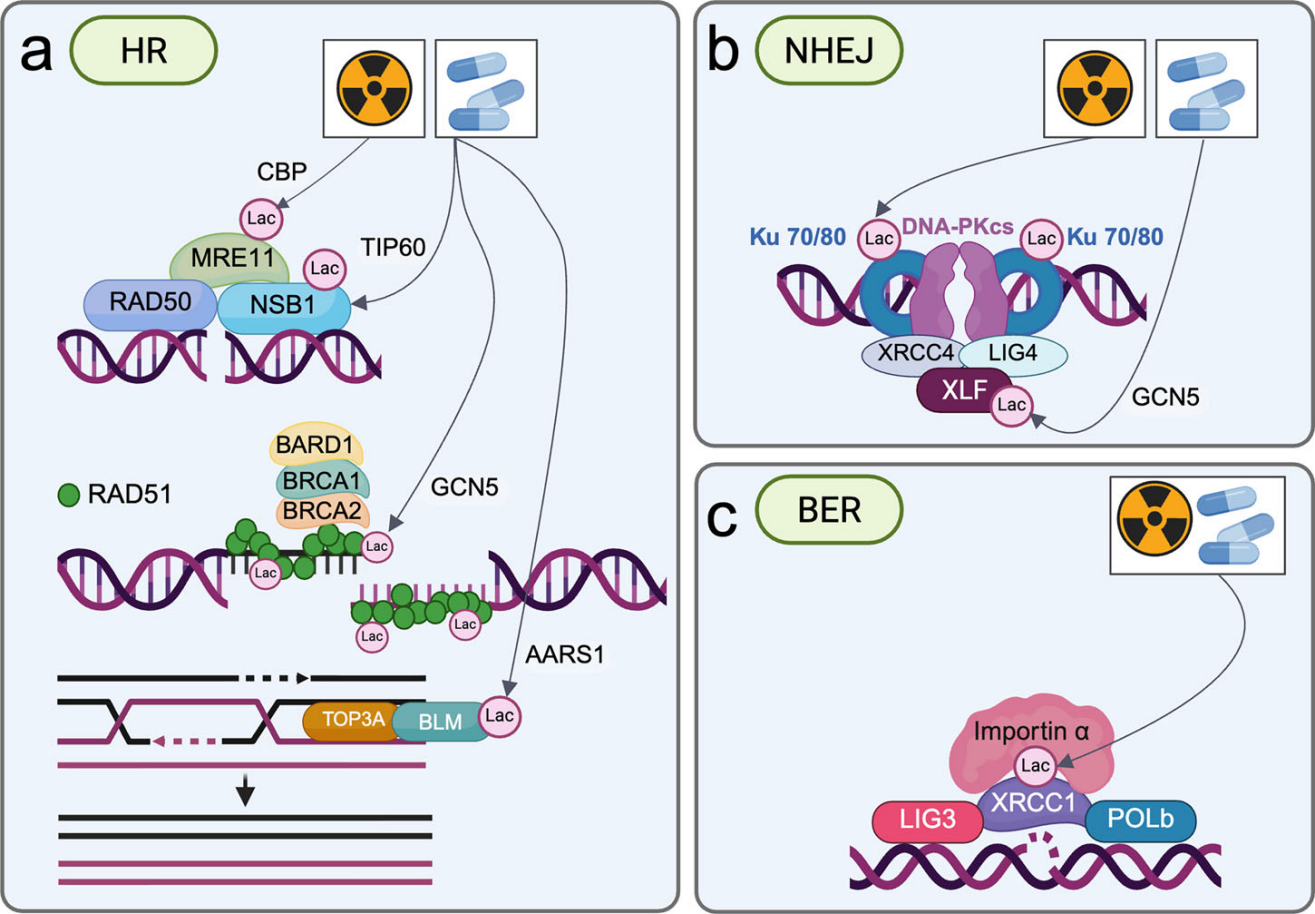
Kla of DNA repair factors in therapy resistance. **a** Kla influences several key steps within the HR repair pathway. Kla of MRE11 and NSB1 by CBP and TIP60, respectively, is required for MRN complex formation, and the accumulation of HR repair proteins at the sites of DNA DSBs, ultimately facilitating DNA end resection and repair. GCN5-mediated Kla of RAD51 enhances HR repair and contributes to therapy resistance. Finally, BLM Kla inhibits its ubiquitination, thereby stabilizing the protein, and strengthening HR activity. **b** Kla of Ku proteins and XLF promotes NHEJ repair and chemoresistance in cancer. Both Ku70 and Ku80 undergo Kla modifications, however, the lactyl transferases responsible for these modifications have not yet been identified. In addition, the functional consequences of Ku protein Kla remain unclear, although it has been associated with radioresistance. Following GCN5 interaction and Kla, XLF shows increased binding to Ku80, resulting in greater XLF recruitment to DSBs and improved NHEJ efficiency. **c** XRCC1 Kla enhances the activity of the BER repair system. Lactylated XRCC1 exhibits an augmented binding affinity for importin α that facilitates its translocation to the nucleus and DNA repair capacity.

In addition, Kla has been reported to reduce the efficacy of DNA-damaging therapies through the direct modification of key factors involved in DNA damage repair and its regulation (Fig. 3).

One of the first discovered targets of protein Kla is the tumor suppressor p53, the guardian of the genome, which, often in conjunction with its family members, transcriptionally controls the DNA damage response [89–93]. Coherently, TP53 is the most mutated gene in cancer [94]. The p53 protein undergoes multiple PTMs, including phosphorylation and ubiquitination [95] that crucially affect its protein stability and transcriptional activity. Zong and colleagues identified p53 as the target of the lactate sensor and lactyl-transferase AARS1, which catalyzes Kla at Lys120 and Lys139 within its DNA-binding domain [96]. In cancer patients harboring wild-type p53, its function is affected by high levels of lactate, correlating with poor survival. In the study, the authors demonstrated that p53 Kla significantly reduces its DNA binding capacity and liquid-liquid phase separation [96]. In tumor patients-derived tissues, the authors observed a significant up-regulation of AARS1, positively correlating with p53 Kla levels, while inversely correlating with p53 activity. Finally, the authors identified β-alanine as a putative inhibitor of AARS1-mediated p53 Kla [96].

Kla of HR-related proteins has been demonstrated in two independent studies [97, 98]. Chen and colleagues reported that, following cancer cells exposure to different DNA damaging agents, MRE11 undergoes CBP-dependent Kla on Lys673 (Fig. 3a). This modification enhances MRE11 DNA binding, thereby facilitating DNA end resection and HR [97]. Coherently, cells expressing the MRE11-K673R mutant exhibited impaired formation of RPA and RAD51 foci. In line with this, sodium lactate treatment increased resistance to cisplatin and olaparib and was associated with a significant reduction in γH2AX foci following DNA damage induction. On the other hand, treatment with CPB- and LDH-inhibitors restored their sensitivity.

In another study, the authors further sustained the importance of Kla in the regulation of HR by focusing on NBS1 modification in gastric cancer cells [98]. In their study, Chen and colleagues identified NBS1 Lys388 as the Kla target of the histone acetyltransferase TIP60 (Fig. 3a). Kla of NBS1 is essential for MRN complex formation and for the recruitment of HR repair components to sites of DNA DSBs. The authors reported that IR treatment of NBS1-K388R-mutant cells delays DNA repair compared to control cells, which is associated with reduced formation of BRCA1 and RAD51 foci. The K388R substitution in the NBS1 protein, which impairs Kla, induces a conformational change that reduces its binding affinity for MRE11. This disrupts proper MRN complex assembly and impairs the recruitment of downstream factors involved DSB repair [98]. Importantly, LDHA was identified among the top upregulated factors observed in the proteomic profile of gastric cancer patients resistant to platinum-based neoadjuvant chemotherapy. Accordingly, LDHA levels positively correlated with the ones of K388 lactylated-NBS1 in resistant patients’ samples, pointing to repair protein Kla as a triggering event in the onset of cancer cell resistance to therapies [98].

The study from Zheng and collaborators [99] corroborated the role of protein Kla in regulating HR via malate enzyme 2 (ME2), which converts glutamine-derived malate to pyruvate, promoting lactate production and Kla levels on MRE11 and NBS1 (Fig. 3a), thereby contributing to chemotherapy resistance in ovarian cancer. Mechanistically, when cancer cells are exposed to cisplatin for extended periods of time to acquire chemoresistance, the availability of glucose transporters diminishes, impairing glucose uptake. The resulting reduction in intracellular glucose triggers ME2 acetylation by increasing and decreasing its association with the acetyltransferase ACAT1 and the deacetylase SIRT4, respectively. ME2 acetylation enhances its enzymatic activity and promotes lactate production via glutaminolysis. These findings linking ME2 acetylation with lactate production and therapy resistance are supported by clinical evidence showing that increased ME2 acetylation is positively associated with reduced responsiveness to platinum therapy and decreased progression-free survival in ovarian cancer patients.

Further investigating platinum resistance in ovarian cancer, Sun and colleagues focused on the regulation of HR by Kla of both histone (see the above paragraph) and non-histone proteins. The upregulation of RAD51 and BRCA2 was associated with GCN5-dependent Kla of H3K9. Furthermore, RAD51 was identified as a protein Kla target (K73la) of the same writer enzyme, further enhancing HR and conferring platinum resistance (Fig. 3a) [77].

Furthermore, increased Kla levels of the HR helicase BLM were detected in different cancer cells, in association with decreased sensitivity to epirubicin. The use of a topoisomerase inhibitor used in cancer therapies markedly reduced BLMK24la levels, thereby increasing cell sensitivity to epirubicin in bladder cancer, breast cancer, and HCC [100]. These findings further confirm the potential effectiveness of targeting Kla of HR factors to overcome resistance.

The NHEJ repair system is regulated by Kla via modification of XLF at K288, a residue located within its Ku-binding motif (Fig. 3b). Kla of XLF enhances its recruitment to DSBs and strengthens the XLF-Ku80 interaction, ultimately resulting in increased NHEJ efficiency. In particular, Jin and co-authors [60] identified GCN5 as the enzyme responsible for XLF Kla in response to DNA damage and demonstrated that GCN5 overexpression increases NHEJ capacity in XLF-wild-type colorectal cancer (CRC) cells, but not in cells expressing the XLF-K288R mutant. The inhibition of LDH or GCN5 impaired XLF loading on chromatin, as well as its interaction with Ku80. Additionally, Ku80-XLF binding was significantly reduced in XLF-K288R mutant cells, thus affecting NHEJ [60].

Given the association of high LDHA and GCN5 expression with poor prognosis in CRC patients, the authors investigated the impact of Kla on chemosensitivity. Inhibition of LDH or disruption of XLF Kla through the K288R mutation sensitized CRC cells to 5-FU treatment, supporting a role for Kla of DNA repair factors in the development of therapy resistance in CRC [60]. A recent report shed light on the mechanism underlying the activation of the NHEJ repair pathway by Kla [101]. The authors reported that, in lung adenocarcinoma (LUAD) cells, LDHA Kla at residues Lys81/Lys318 enhances its enzymatic activity, with AARS1 acting as the lactyltransferase, thus increasing overall cellular Kla. In this study several proteins involved in NHEJ, including Ku70 and Ku80 emerged as additional targets of protein Kla (Fig. 3b). As observed for other repair factors, their Kla markedly enhances the assembly of the repair complex at DNA lesion, leading to hyperactivation of NHEJ and reduced sensitivity of cancer cells to therapy. Coherently, inhibition of LDHA Kla decreases global protein Kla and sensitizes LUAD cells to cisplatin by affecting the activation of the NHEJ repair pathway. This mechanism exemplifies how metabolic reprogramming and Kla mutually influence each other to regulate the therapy sensitivity of cancer cells.

An alternative Kla-dependent mechanism regulating DNA repair has been reported in GBM in response to radiation and chemotherapy [73]. The authors observed a significant increase in XRCC1 Kla levels following sodium lactate treatment and identified Lys247 as the specific modification target (Fig. 3c). The expression of the delactylated XRCC1 mutant (K247A/K247R) was associated with higher levels of DNA damage following radiation and TMZ treatment. Importantly, Li and colleagues demonstrated that the affinity of XRCC1 for the nuclear transport protein importin α is enhanced by the Kla of its Lys247. XRCC1 nuclear import is consequently increased, thus boosting DNA damage repair. Given the key role of XRCC1 in BER and SSBR, enhanced Kla-dependent nuclear import increases its recruitment to the sites of DNA damage. The subsequent upregulation of DNA break repair would ultimately favor chemoresistance in GBM [73].

### Protein lactylation beyond histones and DNA repair factors in therapy resistance

Recent studies have shown that Kla also contributes to chemotherapy resistance via mechanisms that do not involve modifications of histones or DNA repair factors. As an example, SUMO2-Lys11 Kla (SUMO2-K11la) has been observed in response to ferroptosis-induced lactate production in LUAD [102]. This Kla blocks the interaction between SUMO2 and ACSL4, thereby preventing ACSL4 sumoylation. Without this modification, ACSL4 undergoes ubiquitination-dependent degradation. Reduced ACSL4 disrupts lipid metabolism and, ultimately, promotes resistance to ferroptosis. In this study, the authors engineered a peptide that competitively inhibits SUMO2-K11la. By preventing this modification, the peptide induces ferroptosis and sensitizes tumor cells to chemotherapeutic treatments, highlighting a potential new therapeutic intervention for LUAD.

Another target of protein Kla involved in the regulation of the sensitivity of tumor cells to therapy is methyltransferase-like 3 (METTL3), an enzyme responsible for RNA N6-methyladenosine (m^6^A) [103–105]. In particular, METTL3 Kla was identified in the context of triple-negative breast cancer (TNBC) cell resistance to the cytotoxic effects of cisplatin, which is typically employed when irreversible cardiotoxicity or drug resistance to the first-line taxane/anthracycline therapy occurs. In this study, RNA m^6^A modification was reported to be induced during the acquisition of cisplatin tolerance in TNBC cells. Concomitantly, increased HDAC2-mediated delactylation leads to a reduction in METTL3 Kla modification in the cisplatin-tolerant state. Reduced METTL3 Kla subsequently facilitates its interaction with Wilms’-tumor-1-associated protein an essential component of the methyltransferase complex that is required for guiding the complex to its mRNA targets. This, in turn, enhances the expression of genes associated with DNA damage repair in TNBC cells during chemotherapy exposure, thereby conferring cisplatin resistance. Pharmacological inhibition of HDAC2 with tucidinostat was found to augment the efficacy of subtherapeutic doses of cisplatin in eradicating TNBC cells by maintaining the METTL3 Kla state. Remarkably, these findings suggest a potential strategy for mitigating the adverse reactions and toxicity experienced by cancer patients receiving platinum-based therapies.

The lipidomic analysis of plasma samples from twenty-two acute lymphoblastic leukemia (ALL) patients has revealed higher levels of sphingomyelin (SM) in the leukemic condition compared to the remission one[106]. Lin and colleagues demonstrated that SM drives ALL progression via glucose-dependent proliferation, enhancing glycolysis and lactate production, thereby promoting Kla. The authors identified Lys14 as the Kla target on Caspase 3 (CASP3) resulting in inhibition of its apoptotic function. Consistently, Kla-dependent inactivation of CASP3 promoted leukemia growth demonstrating an additional Kla-mediated mechanism of cancer resistance to apoptosis [106].

While investigating mechanisms of resistance to the TKI levatinib in HCC, Liu and co-authors identified aldo-keto reductase family 1 member B10 (AKR1B10) as a Kla substrate of the lactyl-transferase AARS1 [107]. Their results show that Kla at Lys173 of AKR1B10 impedes its ubiquitination and subsequent degradation, thereby increasing its protein stability. Moreover, AKR1B10-K173la interacts with LDHA and facilitates its phosphorylation, boosting lactate production, which, in turn, drives H3K18la-mediated transcriptional upregulation of LDHA, creating a positive feedback loop that drives resistance to levatinib in HCC cells [107].

Zheng and co-authors investigated changes in mitochondrial dynamics, which contribute to EGFR-TKI resistance in lung cancer and are associated to metastasis [108]. The authors reported death-associated protein kinase 2 (Dapk2) dysfunction as a key driver of mitochondrial metabolic reprogramming and structural remodeling. Specifically, the authors observed that EGFR-TKI-resistant cells display a significantly higher resistance to anoikis, associated with the downregulation of Dapk2. Loss of Dapk2 reduced phosphorylation (Ser34) and promoted Kla (Lys282) of the mitochondrial protein Mic60, thereby remodeling mitochondrial cristae and enhancing OXPHOS. The mitochondrial metabolic changes correlated with increased resistance and metastatic potential in mouse models. These findings highlight the role of Kla in the reprogramming of mitochondrial metabolism, which drives resistance and promotes metastasis in lung cancer [108].

Focusing on ferroptosis resistance in CRC, Yang and colleagues conducted a drug screening study, combining multiple chemotherapeutic and immunotherapeutic drugs with the ferroptosis inducer RSL3 [109]. Their findings revealed a significant sensitization effect of the HDAC inhibitor vorinostat toward ferroptosis. They discovered elevated Kla levels on HDAC1 Lys412, which were significantly reduced by vorinostat. Mechanistically, the reduction of HDAC1 K412la suppressed HDAC1 expression and increased H3K27 acetylation. Consequently, the m6A demethylases FTO and ALKBH5 were transcriptionally upregulated and lowered the m6A modification and stability of FSP1 mRNA. Reduced FSP1 expression finally enhanced CRC sensitivity to ferroptosis [109]. These findings further corroborate the notion that resistance to ferroptosis in CRC is strongly supported by Kla.

### Role of lactylation in immunotherapy resistance

Growing evidence has revealed a complex functional interplay between lactate, Kla modifications, and immune regulation within the TME. In general, lactate accumulation in the TME and the resulting Kla events promote immune evasion, thereby reducing the effectiveness of immunotherapies. In syngeneic mouse models of melanoma and CRC, modulation of the lactate transporter MCT4/SLC16A3 increases lactate levels in the TME, which, in turn, reinforces immunosuppressive conditions by promoting the accumulation of regulatory T cells (Tregs) and myeloid-derived suppressor cells (MDSCs) [110]. Consistently, in PDAC, genetic disruption of the predominant bicarbonate transporter SLC4A4 reduces glycolytic activity and lactate production by tumor cells, leading to enhanced CD4⁺ and CD8⁺ T cell–mediated immune responses [111]. Notably, in melanoma, extracellular lactate acts as a key driver of dendritic cell (DC) dysfunction by activating an SREBP2-dependent transcriptional program that promotes the development of CD63⁺ mature regulatory DCs (mregDCs) within the TME [112].

**Fig. 4:**
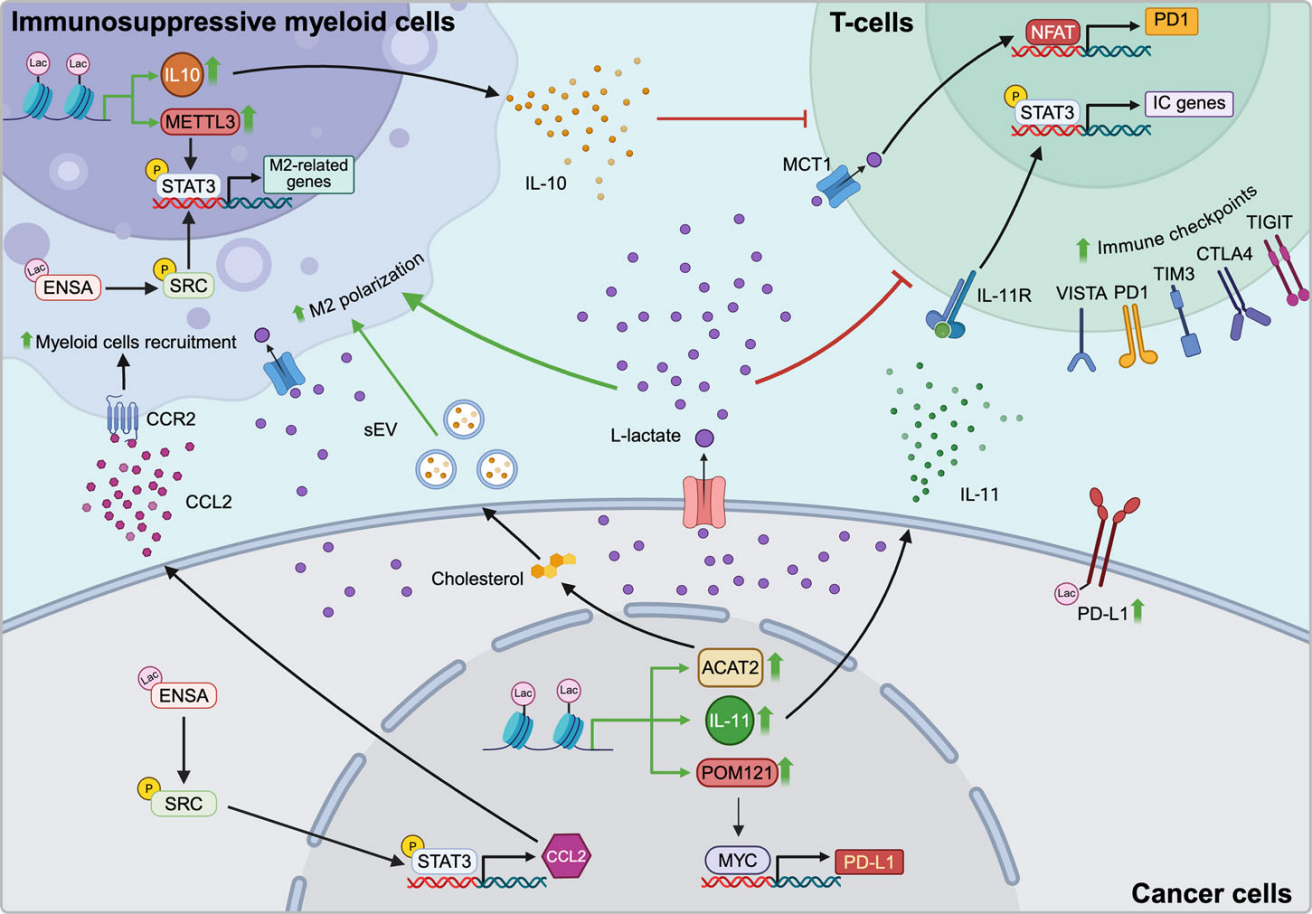
Lactate-mediated immunosuppression in the TME. L‑lactate shapes an immunosuppressive TME by promoting the recruitment and functional reprogramming of immunosuppressive myeloid cells, while selectively impairing T effector cells and favoring T regulatory cell persistence. Lactate‑driven Kla enhances the expression and stabilization of immune checkpoint molecules in both cancer cells and T cells. In addition, it promotes M2-like macrophage polarization, thus further contributing to suppressing anti-tumor immunity and promoting tumor progression.

Beyond its role in modulating T cell-mediated immune responses, extracellular lactate also plays a critical role in regulating myeloid cell differentiation, including MDSCs and tumor-associated macrophages (TAMs). Lactate accumulation within the TME promotes polarization of TAMs toward an M2-like phenotype, accelerates MDSC expansion, and suppresses natural killer cell-mediated cytotoxic activity [113, 114]. In myeloid cells, NOTCH-dependent suppression of the lactate transporter MCT2 reduces intracellular lactate levels, triggering signaling cascades that favor TAM polarization toward the pro-inflammatory M1 phenotype [115], reinforcing the notion that lactate favors an immunosuppressive phenotype (Fig. 4). Interestingly, D-lactate has been reported to reprogram M2-like TAMs toward an M1 phenotype and remodel the immunosuppressive TME in HCC, suggesting that distinct lactate isomers may exert divergent effects on tumor immune regulation [116].

As described above, lactate serves as a key donor for protein Kla through its metabolic intermediate lactyl-CoA. Similar to its role in radio- and chemotherapy response signaling, histone Kla, particularly at H3K18, has emerged as an important epigenetic modification regulating immune suppression across multiple tumor types (Fig. 4). For example, in non-small cell lung cancer (NSCLC), H3K18la promotes PD-L1 expression by facilitating MYC nuclear translocation and its direct binding to the PD-L1 gene promoter [117]. H3K18la has also been implicated in M2 macrophage polarization through transcriptional regulation of acetyl-CoA acetyltransferase 2 (ACAT2) [118]. Consistently, H3K18la enhances the expression of METTL3 in tumor-infiltrating myeloid cells [119], and increased METTL3 expression activates the JAK1/STAT3 signaling axis, thereby reinforcing the immunosuppressive functions of these cells (Fig. 4) [119].

In addition to H3K18la, H3K9la has also been linked to immune evasion. In head and neck squamous cell carcinoma (HNSCC), H3K9la promotes interleukin-11 (IL-11) expression, which in turn activates immune checkpoint gene expression in CD8⁺ T cells via JAK2/STAT3 signaling (Fig. 4) [120]. In GBM, elevated intracellular lactate levels produced by a subset of highly glycolytic monocyte-derived macrophages drive histone Kla, leading to suppression of T cell activity through increased IL-10 expression (Fig. 4) [121].

Beyond histones, Kla of non-histone proteins also contributes to shaping the TME and promoting immune evasion. In PDAC, a tumor type characterized by high lactate accumulation and an immune-cold phenotype, Kla of endosulfine-α activates STAT3/CCL2 signaling, thereby enhancing TAM recruitment and fostering an immunosuppressive microenvironment (Fig. 4) [122, 123]. Moreover, the immune checkpoint protein PD-L1 has been reported to undergo Kla, an event that delays its protein degradation, thereby increasing its levels (Fig. 4) [124]. In addition to increase the expression of PD-L1, lactate has also been shown to regulate PD-1 expression in Tregs through an NFAT1-dependent mechanism [125]. Collectively, these findings support the concept that lactate functions as an important metabolite controlling immune checkpoint expression within highly glycolytic TMEs, potentially influencing responsiveness to PD-1 blockade therapies [125].

Accordingly, a significant association between elevated protein Kla levels and poor patient prognosis has been reported in multiple tumor types. In HNSCC, NSCLC, and PDAC, global or histone-specific protein Kla correlates with reduced responsiveness to immunotherapy [111, 117]. These findings indicate that therapeutic strategies modulating lactate metabolism may synergize with immune checkpoint inhibition to improve outcomes in solid tumor. Supporting this hypothesis, most of the available evidence has been generated using mouse tumor models. Notably, a phase I clinical study involving twenty patients with advanced solid tumors reported that a serine/glycine-free diet, a nutritional condition that enhances the glycolytic flux and promotes PD-L1 Kla, led to elevated expression of granzyme B and interferon-γ in both CD4⁺ and CD8⁺ T cells. Although confirmatory studies are necessary, this evidence suggests that modulation of lactate metabolism may be instrumental in shaping systemic antitumor immunity [124].

Beyond the canonical K_L-la_, K_D-la_ has also been involved in shaping the TME, mainly through the modulation of the inflammatory signaling. In the absence of GLO2, RAW264.7 macrophages exhibit elevated LGSH levels and increased D-Kla of histone H3 at Lys79 and Lys18. These epigenetic modifications enhance the expression of pro-inflammatory genes, thereby amplifying inflammatory responses upon exposure to inflammatory stimuli [27]. The role of K_D-la_ in inflammation has also been described in an independent study, although with contrasting outcomes [29]. By exploiting in vitro and in vivo mouse models, this study demonstrated that inflammation-mediated downregulation of GLO2 promotes K_D-la_ of several transcription factors involved in the inflammatory signaling, including NF-κB. Notably, D-lactylated NF-κB exhibits reduced transcriptional activity, suggesting that Kla may contribute to the attenuation of inflammatory responses.

Although reporting contrasting functional outcomes, these studies lay the foundation for future investigations aimed at elucidating the contribution of D-Kla to immune regulation and TME remodeling, particularly in those tumors characterized by highly inflammatory phenotypes. Furthermore, the distinct effects exerted by lactate isomers on macrophage polarization and immune evasion [23] highlight the need for deeper exploration of isomer-specific Kla events and their impact on immune regulation within the TME.

In conclusion, multiple evidence highlights an intricate interplay between lactate metabolism, Kla events, and immune regulation, paving the way for future investigations aimed at leveraging this functional axis to enhance the efficacy of cancer immunotherapy.

## Potential of lactylation as a therapeutic target

Accumulating evidence indicates that Kla modulation, when combined with radio- chemo- and immunotherapy may represent a promising strategy to overcome therapeutic resistance in tumors.

By exploiting a cell-penetrating peptide capable of inhibiting MRE11 Kla, Chen and collaborators showed that this approach restored HR activity and increased the sensitivity of CRC cells to cisplatin and PARP inhibitors [97].

Blocking the endogenous lactate production using either the glycolysis inhibitor 2-deoxy-D-glucose or LDH inhibitors (LDHi) is emerging as a potential therapeutic strategy to increase tumor cell sensitivity to anticancer therapies.

Although clinical trials are needed to assess the safety and potential efficacy of LDHi in cancer treatment, preclinical studies have shown that daily administration of the LDHi, sodium oxamate, in mice effectively suppressed tumor growth with minimal toxicity [97, 126]. The anticancer effect of oxamate has also been reported in a humanized model of lung cancer [127]. This study also reported the effectiveness of oxamate in potentiating the antitumor action of the immune checkpoint inhibitor pembrolizumab.

MCT inhibitors are also under investigation due to their ability to block lactate intracellular transport. There is evidence that these compounds can restore the sensitivity of cancer cells to both radio- and chemotherapy [128, 129].

Stiripentol (Diacomit®), an antiseizure medication used clinically, has been shown to be effective in overcoming chemotherapy resistance of GBM cells to TMZ in vivo [98]. In addition, Stiripentol was found to overcome cisplatin resistance in mice bearing patient-derived xenografts of chemotherapy-naive gastric cancers, by preventing NBS1 K388 Kla [98].

In in vivo models, D34-919, a drug that disrupts the ALDH1A3/PKM2 interaction, enhanced GBM cell sensitivity to chemoradiotherapy by reducing lactate production, with no adverse effects on body weight or organ integrity [73]. Notably, D34-919 may exert minimal effects on the metabolic function of normal cells, potentially resulting in lower toxicity compared with traditional PKM2 inhibitors.

Additional compounds that indirectly prevent Kla, such as K-604, which suppresses ME2 enzyme activity, and the HDAC inhibitors tucidinostast and vorinostat, were found to enhance chemosensitivity in ovarian [99], CRC [109] and breast cancer cells [103], respectively. The study by Zong and colleagues reported that β-alanine is able to disrupts lactate binding to AARS1 by competing with lactate for enzyme binding, thus reducing p53 Kla, and inhibiting tumor growth in mouse models of breast cancer [96]. However, the authors did not evaluate the effect of β-alanine on tumor cell response to anticancer therapies. Further investigations into the impact of β-alanine on the therapeutic responses would be of significant interest in the field.

Alternative strategies can be employed to directly target Kla substrates to counteract therapy resistance. For instance, AKR1B10 can be inhibited with epalrestat to overcome resistance to lenvatinib in preclinical models of HCC [107].

## Conclusions and future perspectives

Kla has emerged as a central mechanism linking metabolic reprogramming to epigenetic regulation and therapeutic resistance in cancer cells. As highlighted in this review, enhanced glycolysis and resulting lactate production fuel histone and non-histone events, including activation of DNA damage repair factors, which promote resistance to anti-cancer therapies. At the chromatin level, site-specific histone marks drive transcriptional programs that sustain DNA repair, thereby contributing to resistance to chemo- and radiotherapy. Beyond histones, Kla directly modifies key modulators of DNA damage repair or proteins that ultimately regulates genome stability, thereby limiting treatment efficacy. Kla also sustains immune suppression within the TME, thus impairing antitumor immune responses and reducing the effectiveness of immune checkpoint blockade.

From a therapeutic viewpoint, targeting Kla-associated enzymes represents a promising therapeutic strategy to overcome therapy resistance. Preclinical studies have shown that inhibition of lactate transport or production and modulation of Kla writers and erasers, as well as disruption of lactate-driven feedback loops can sensitize tumors to conventional therapies and enhance the efficacy of immunotherapy. Nevertheless, key challenges remain to be addressed, including the partial identification of Kla-regulating enzymes, the effects of distinct Kla isomers, and the interplay between Kla and other PTMs. Further mechanistic and translational studies will be fundamental to exploit Kla-targeted strategies for improving cancer treatment outcomes.
